# Decline of six native mason bee species following the arrival of an exotic congener

**DOI:** 10.1038/s41598-020-75566-9

**Published:** 2020-10-30

**Authors:** Kathryn A. LeCroy, Grace Savoy-Burke, David E. Carr, Deborah A. Delaney, T’ai H. Roulston

**Affiliations:** 1 Department of Environmental Sciences, University of Virginia, Charlottesville, VA USA; 2 Department of Entomology and Wildlife Biology, University of Delaware, Newark, DE USA

**Keywords:** Conservation biology, Invasive species, Entomology

## Abstract

A potential driver of pollinator declines that has been hypothesized but seldom documented is the introduction of exotic pollinator species. International trade often involves movement of many insect pollinators, especially bees, beyond their natural range. For agricultural purposes or by inadvertent cargo shipment, bee species successfully establishing in new ranges could compete with native bees for food and nesting resources. In the Mid-Atlantic United States, two Asian species of mason bee (*Osmia taurus* and *O. cornifrons*) have become recently established. Using pan-trap records from the Mid-Atlantic US, we examined catch abundance of two exotic and six native *Osmia* species over the span of fifteen years (2003–2017) to estimate abundance changes. All native species showed substantial annual declines, resulting in cumulative catch losses ranging 76–91% since 2003. Exotic species fared much better, with *O. cornifrons* stable and *O. taurus* increasing by 800% since 2003. We characterize the areas of niche overlap that may lead to competition between native and exotic species of *Osmia*, and we discuss how disease spillover and enemy release in this system may result in the patterns we document.

## Introduction

International trade creates opportunities for plant and animal species to be intentionally or inadvertently introduced into novel ecosystems where they may interact with native species. One outcome of species introductions is the potential for competitive interactions with native species, especially those that are most closely related to the introduced species. When closely related species co-occur and overlap in key parts of their life cycle, negative competitive interactions may result, potentially altering ecological and evolutionary trajectories^1^. The success of exotic species establishing in their new range may be facilitated by several factors, including escaping enemies from its home range^2^ and introducing novel enemies, such as exotic diseases, to native competitors in the new range^3^.

Bees (Hymenoptera: Anthophila) are a group of insects with great potential for evaluating the impacts of introduced species on native species, both as perpetrators and casualties, due in part to their anthropogenic associations. Bee species have been intentionally shipped around the world for agricultural pollination and become naturalized in novel environments^4–7^. In addition to intentional introductions, other bee species have been accidentally transported to novel environments via cargo that they nest inside, including wooden packing crates^8^ and lumber for furniture-making^9^. In North America alone, at least thirty-nine exotic bee species have become naturalized, with many spreading extensively since they were first observed^10^ but no solitary species have been extensively monitored for impacts.

Ecological effects of introduced exotic bees on native bees have been hypothesized^7,11^, and studies have recorded range contractions, reduced abundance, or the complete disappearance of native bee species following introductions of social honey bees and bumble bees for agricultural pollination^12–14^. However, of the exotic bee species known to occur in North America, the majority are from the solitary, cavity-nesting family Megachilidae^10,15^. At present, there is little understanding about the impacts of the establishment and spread of introduced solitary bee species on local native bee populations^16,17^. There has been little monitoring of native megachilid communities to determine their population trajectories in the added presence of closely-related exotic competitors.

**Figure 1:**
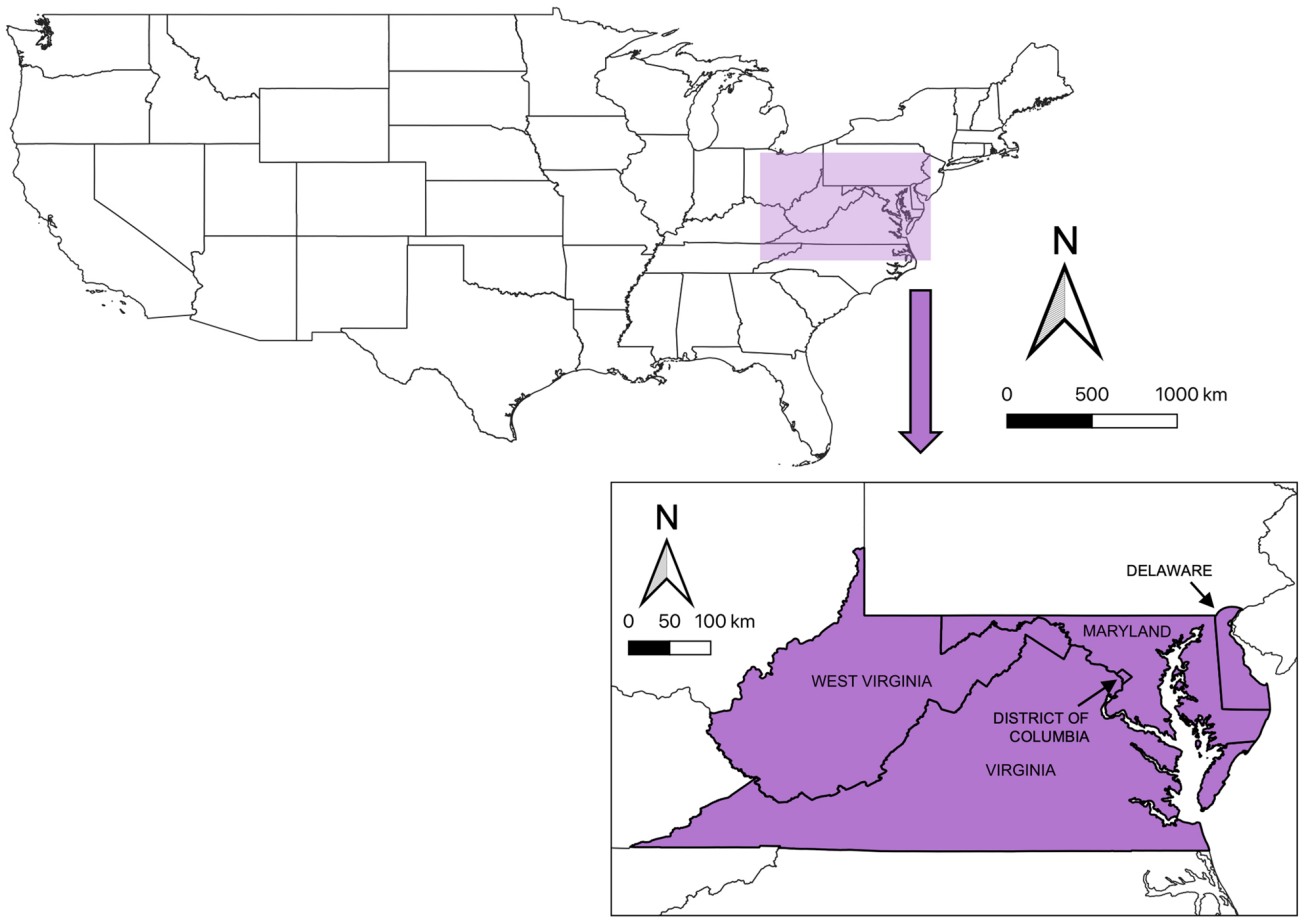
Map of the continental United States of America. Shaded box designates general sampling area of the Mid-Atlantic Region, with inset map depicting states and territory where sampling events were conducted.

In North America, the megachilid genus *Osmia* consists of cavity-nesting bees called “mason bees” with flight periods in the spring and early summer, with great chances of phenological overlap among congeners in activities such as foraging and nesting. Approximately twenty *Osmia* species are native to the Mid-Atlantic United States (Fig. 1)^18^. In addition to native *Osmia*, two mason bee species introduced from Asia have recently naturalized in the region. The Japanese horn-faced bee, *Osmia cornifrons* Radoszkowski, was intentionally introduced from Japan by the United States Department of Agriculture (USDA) in the 1970s for crop pollination services^19^. In 2002, another mason bee from Asia, *Osmia taurus* Smith, was first documented in the United States, without record of its being intentionally imported^20^.

With multiple introductions of *Osmia* species to North America, this group is well-suited for examining impacts of exotic species on a community of native congeners. This study sought to evaluate any changes in abundances of native and exotic *Osmia* species over thirteen years in the Mid-Atlantic region of the United States following the detection of the second introduced mason bee species, *Osmia taurus*.

## Methods

### Study system

Bees in the genus *Osmia* (Family Megachilidae) are solitary, cavity-nesting species. They are referred to as “mason bees” for their mason-like use of mud, masticated leaf pulp, or other substrates to partition brood cell chambers and seal nest entrances. *Osmia* in North America are generally univoltine and commonly active mainly in spring to early summer in temperate North America^21^.

### Data collection

A combined, long-term ecological monitoring dataset of bees (Hymenoptera: Anthophila) was used to evaluate changes in raw abundance of springtime *Osmia* (Family Megachilidae) species. This data set was contributed by authors G.S.-B. and D.A.D. (“Delaware dataset”), a network of citizen science program participants coordinated by Sam Droege at the Patuxent Wildlife Refuge (Beltsville, MD USA) (“Patuxent dataset”), and a network of citizen science program participants coordinated by authors K.A.L. and T.H.R. at Blandy Experimental Farm (“Blandy dataset”). The most concentrated sampling efforts occurred in the Mid-Atlantic United States of Delaware, Maryland, Virginia, and West Virginia, as well as the District of Columbia (Fig. 1). These sampling events were conducted by citizen science program participants and research coordinators, and sampling events followed a general pan-trapping protocol^22,23^ in which plastic bowls filled with a preservative trapping medium were placed in open landscapes to catch flying invertebrates.

Trapping medium consisted of either soapy water or a 50:50 water to propylene glycol solution. Bowl sizes within our sampling events were equally sized but ranged from a volume capacity of 3.25 oz. up to 16 oz. across sampling events, with over 95% of sampling events using 3.25 oz., 3.5 oz., or 12 oz. bowl sizes. Previous research has found no significant relationship between bowl size and number of bees caught in pan-trapping using containers up to 12 oz.^24,25^, but greater bee catch abundance has been observed using 20 oz. bowls compared to smaller-sized bowls^26^. The number of bowls deployed in a given sampling event ranged from three to 250, with a median of nine bowls. The duration of sampling events ranged from one to 50 days, with a median of seven days. Collected specimens were cleaned, pinned, entered into a database, identified, and verified by Sam Droege to the lowest taxonomic category possible (species in most cases). This region of the Mid-Atlantic United States is where the exotic *Osmia cornifrons* was intentionally introduced^19^ and where another exotic species (*O. taurus*) was first recorded^20^.

### Data filtering

Prior to analyses, we extracted from the combined dataset all regional records of *Osmia* that were identified to species (or to genus if unable to classify to species) and contained complete location information (latitude and longitude coordinates). We limited our dataset to years in which all specimens captured within each sampling event were identified. These years were 2003–2015 inclusively for the Patuxent data set, 2014 and 2015 for the Delaware dataset, and 2017 for the Blandy dataset. Further, we examined all commentary fields in the dataset and excluded all sampling events that noted inclement weather. We did this in order to reduce the likelihood of failed captures being scored as records of absence when they might actually represent poor conditions for sampling. Because the colors of bowls used in pan-trapping surveys can impact their attractiveness to bees and therefore impose a detection bias^27^, sampling events were excluded if they did not deploy all three of the most effective colors known to collectively attract the most bees, including *Osmia*: fluorescent blue, white, and fluorescent yellow^24^.

We also chose to exclude one sampling event from the Patuxent dataset in 2010 in which 548 individual *Osmia* were captured in a single day, the largest catch in the combined dataset (the next largest filtered sampling event captured only 85 individuals over 30 days). Of the 548 specimens in that single sample, 539 were identified as *Osmia taurus*. With concern that this specific sampling event may have been conducted directly by a large nesting aggregation of *O. taurus*, in such a way that other sampling events were not, we chose to exclude this sampling event from our data set (but see Supplementary Table S1 for estimate of *O. taurus* with inclusion of this outlier). Finally, in order to maximize our ability to detect trends within species over time, only *Osmia* species with greater than 50 specimen records were selected for species-level analysis (Table 1). *Osmia* species with greater than 50 specimen records included six native species (*Osmia atriventris* Cresson; *O. bucephala* Cresson; *O. collinsiae* Robertson; *O. georgica* Cresson; *O. lignaria* Say; and *O. pumila* Cresson) and two exotic species (*O. cornifrons* Radoszkowski, and *O. taurus* Smith).

Overall, along with removing 548 *Osmia* specimens from the single sampling event in 2010 described above, we also removed 14 sampling events (consisting of 47 *Osmia* specimens) due to bad weather, and another 355 sampling events (consisting of 3021 *Osmia* specimens) were excluded due to not employing all three bowl colors simultaneously. In grand total, 370 sampling events with 3616 *Osmia* specimens were excluded from all datasets, and a remaining 5901 *Osmia* specimens from 1125 sampling events were used for analyses.

### Spatial variation in sampling effort

Sampling events were not uniformly distributed across space. In order to reduce the potential for geographic bias in sampling intensity to distort perceived temporal patterns, we combined nearby sites through a process of spatial clustering. We used the known average female intertegular (IT) span for each of the eight *Osmia* species analyzed in this study to calculate an average typical foraging distance using previously published parameters^28^. Across the eight species, the calculated typical foraging distances ranged from 0.22 km (*O. pumila*), up to 1.72 km (*O. bucephala*). The average typical foraging distance of all eight *Osmia* species was estimated at 0.63 km. We decided to treat 0.63 km as a foraging radius, and we used 1.2 km (roughly twice the foraging radius) as a threshold distance for clustering two sampling sites. Thus, if two sampling events occurred within 1.2 km of each other, those sampling events were assigned to a common spatial cluster. This was done iteratively until all sampling events were assessed. After all sampling events were subjected to this clustering process, any cluster that contained sampling events spanning a total geographic distance greater than 1.2 km were then subjected to an algorithm termed affinity propagation, which broke down these larger clusters into a calculated number of smaller clusters based on the relative distances of sites within each cluster^29^.

Sampling events occurring within the same year and assigned to the same spatial cluster had their catch abundance data pooled. In this manner, spatial cluster-year is used as a random effect in the statistical analysis to control for the uneven spatial structure of the dataset to the extent of typical foraging distances. From 1125 sampling events occurring from 2003 to 2017, this process produced a total of 398 spatial cluster-years from 298 spatial clusters.

### Temporal variation in sampling effort

Sampling events varied in the number of bowls used and event duration, which in turn can impact bee captures and therefore detection likelihood^30^. In order to account for this variation in sampling effort, the number of days of each sampling event and the number of bowls deployed for each sampling event were multiplied together to produce “bowl days.” These bowl days were then summed within each spatial cluster-year, producing an aggregate sampling effort metric. Compared to alternative measures, this metric was found to be the strongest predictor explaining the abundance of *Osmia* captured in sampling events (specimen abundance = -18.88709 + 7.71731*aggregate sampling effort, F (1378) = 74.18, *p* < 0.0001, R^2^ = 0.1646) and thus it was included as an offset variable in all statistical analyses examining capture abundance over time. Within-year variation in sampling effort was also evaluated (see Supplementary Information Methods).

### Statistical analyses

The change in abundance over time was modeled both for the pooled dataset of all *Osmia* records and for each *Osmia* species (with n > 50) independently using Generalized Linear Mixed Models in SAS 9.4 with PROC GLIMMIX. Raw abundance for each spatial cluster-year was the dependent variable, and spatial cluster was specified as a random effect, with a random intercept specified for spatial cluster. Year was specified as a fixed effect, and the log-transformed aggregate sampling effort metric (“bowl days”) for each spatial cluster-year was used as an offset variable. The appropriate model response distribution was selected for each species (assessed by evaluating residuals and fit statistics from models using the Poisson, generalized Poisson^31^, and negative binomial distributions), all of which followed a negative binomial distribution (Table 1). All models were specified with a log link function.

Analyses at the level of genus *Osmia* were performed two ways, one including only records from species that had greater than 50 specimens, and the second including all specimen records regardless of the quantity per species or whether they were identified to the species level. For each species and genus-wide model, we inspected the standardized residuals of each model for any remaining spatial autocorrelation using bubble plots, correlograms, variograms, and calculation of Moran’s I. We additionally ran these analyses at larger scales beyond 1.2 km: we conducted analyses following clustering at 3 km, 5 km, 10 km, and 20 km. Lastly, as a more conservative analysis, we performed analyses at 1.2 km using only clusters that included at least two years of sampling.

## Results

**Figure 2:**
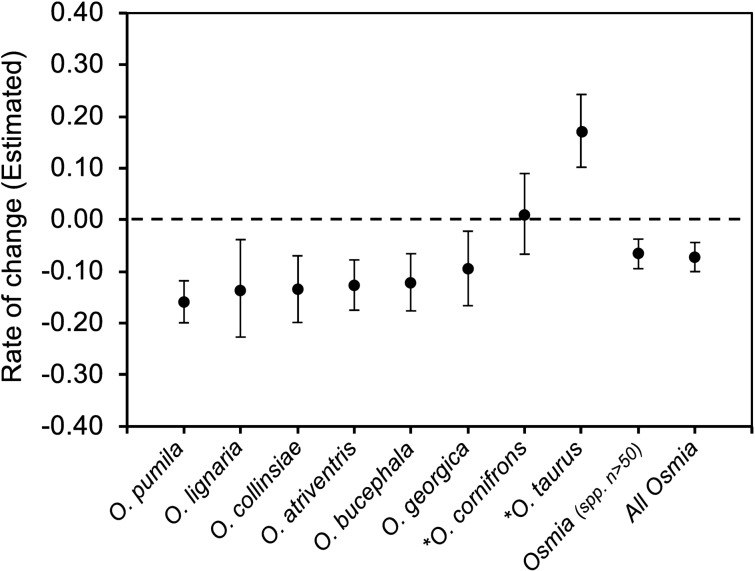
Estimated rates of yearly change for models of each species-specific and genus-wide analysis performed, with 95% confidence intervals. Estimates in original scale (reverse i-link) are displayed. Species labeled with an asterisk (*) are exotic to North America.

Overall, 5901 specimen records from 1125 sampling events were analyzed. Annual capture rates for the genus *Osmia* as a whole significantly decreased at a mean of 6.64% per year for the species with n > 50 specimens, and declining at a mean of 7.25% per year when all *Osmia* records were included in the dataset (Fig. 2, Table 1). All six native *Osmia* were found to be significantly declining in mean raw abundance, ranging from a 9.70% yearly mean decline (*O. georgica*) to a 15.97% yearly mean decline (*O. pumila*). The exotic *O. taurus* was found to be significantly increasing at a magnitude of 16.99% per year (Fig. 2, Table 1). The estimated rate of change for exotic and commercially managed *O. cornifrons* was not significantly different from zero (Fig. 1, Table 1). There was no change in within-year sampling effort across years (Supplementary Table S2, Supplementary Fig. S1).

**Table 1:** Results output from models for each species-specific and genus-wide analysis performed. Estimates are given on original scale. Species-specific models must have had greater than 50 specimens to be considered for analyses. NB = Negative Binomial distribution selected for analysis (as described in Methods section).

Species or Genus	Native/Exotic	*n*	Model	Estimated mean change per year	SE	*P*	Percent mean change per year
*Osmia atriventris*	Native	442	NB	**0.8723**	**0.0245**	** < .0001**	− **12.77**
*Osmia bucephala*	Native	217	NB	**0.8774**	**0.0279**	** < .0001**	− **12.26**
*Osmia collinsiae*	Native	135	NB	**0.8634**	**0.0325**	**0.0002**	− ** 13.66**
*Osmia cornifrons*	Exotic	618	NB	1.0084	0.0393	0.8310	− 0.840
*Osmia georgica*	Native	293	NB	**0.9030**	**0.0363**	**0.0126**	− **9.70**
*Osmia lignaria*	Native	76	NB	**0.8622**	**0.0475**	**0.0082**	− **13.78**
*Osmia pumila*	Native	1588	NB	**0.8403**	**0.0205**	** < .0001**	− **15.97**
*Osmia taurus*	Exotic	2288	NB	**1.1699**	**0.0355**	** < .0001**	** + 16.99**
*Genus Osmia (n* > *50)*	Both	5657	NB	**0.9336**	**0.0144**	** < .0001**	− ** 6.64**
*Genus Osmia (all)*	Both	5901	NB	**0.9275**	**0.0142**	** < .0001**	− ** 7.25**

Boldface indicates models with significance at *p* < 0.05.

**Figure 3:**
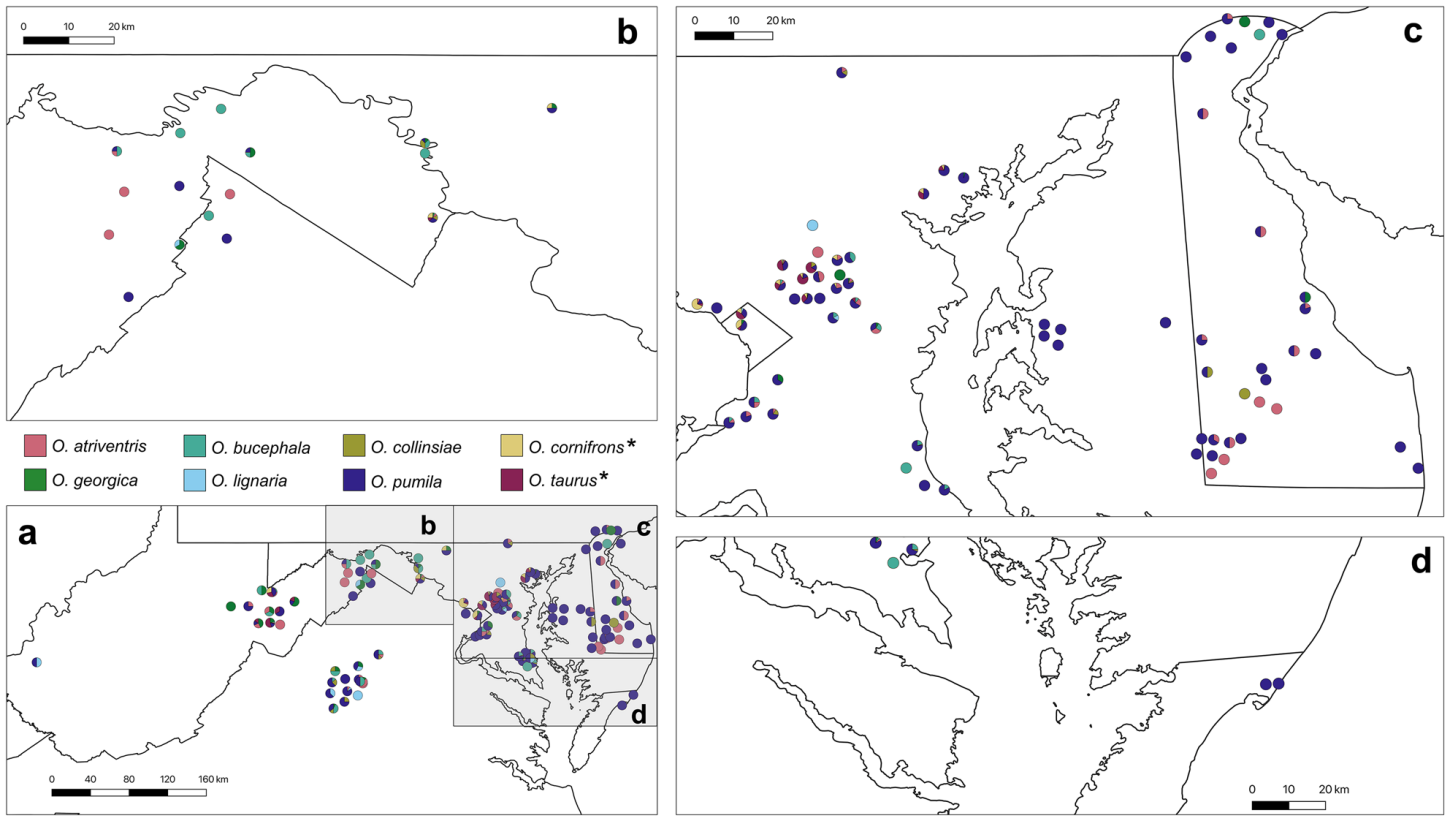
Mason bee (genus *Osmia*) composition of sites in the Mid-Atlantic Region of the United States, 2003–2009. Pie charts at each site represent the proportions of *Osmia* species according to color legend. Placement of pie charts represent approximate location of sampling site. Bee species names accompanied with an asterisk (*) are exotic to the Mid-Atlantic United States.

**Figure 4:**
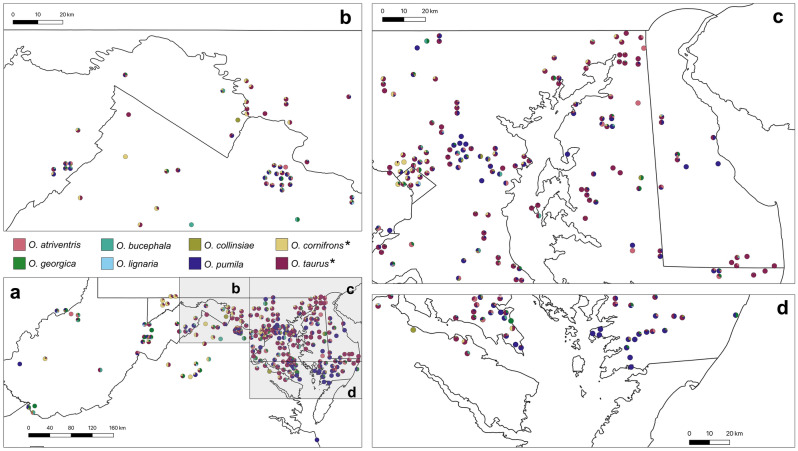
Mason bee (genus *Osmia*) composition of sites in the Mid-Atlantic Region of the United States, 2010–2017. Pie charts at each site represent the proportions of *Osmia* species according to color legend. Placement of pie charts represent approximate location of sampling site. Bee species names accompanied with an asterisk (*) are exotic to the Mid-Atlantic United States.

Residual spatial autocorrelation was found only for *O. pumila*, such that sites at close distances were more similar (Supplementary Table S4). Geographic breakdowns of *Osmia* species occurrence are provided in Fig. 3 for 2003–2009 and Fig. 4 for 2010–2017. Using greater clustering thresholds of 3 km, 5 km, 10 km, and 20 km, we found that all native *Osmia* species remained in significant decline, *O. taurus* remained significantly increasing, and *O. cornifrons* did not change significantly over time (Supplementary Fig. S2). From analysis of only clusters with multiple sampling years, *O. taurus* remained significantly increasing as in the full analysis, *O. cornifrons* remained insignificant in its estimated change, and all six analyzed native species still were estimated to be experiencing yearly declines (Supplementary Fig. S3, Supplementary Table S5), all of which remained significant with the exception of *O. lignaria* for which statistical power was reduced (only 41 specimens for *O. lignaria* in multi-sampled clusters). However, the estimate of decline (0.9006) remained similar to estimated decline of *O. lignaria* in the main analysis (0.8622) (Supplementary Table S5, Table 1). The landcover types of sampling locations in the early years of the study did not differ from the landcover composition sampled later in the study (Supplementary Table S6).

## Discussion

Dialogue concerning insect declines in the Anthropocene often fails to tease apart the insect “winners” and “losers”^32^. We report a significant surge in the abundance of the exotic solitary bee species *Osmia taurus* with concurrent losses of all six native *Osmia* species in our analysis. As a percentage of all *Osmia* captured, exotic *O. taurus* increased from approximately 22% of captures in 2003–2009 to being the most commonly caught *Osmia* species with over 43% of all captures in 2010–2017. Thus, despite exotic *O. taurus* being first recorded in the U.S. only in 2002^20^, it has now become the most commonly caught *Osmia* species in the region by far. Given that *O. cornifrons* was intentionally introduced, actively propagated, and shipped around the region at least two decades before *O. taurus* arrived, it is surprising that *O. taurus* has gone from a first record in 2002 to being more commonly collected than *O. cornifrons* in every year since 2009. It does not appear to be a case of mistaken identity, as we have examined regional museum specimens to ascertain that *O. taurus*, which is superficially similar to *O. cornifrons*, wasn't mistakenly identified by researchers as *O. cornifrons* prior to knowledge of the arrival of *O. taurus* in the region (K.A.L. and S. Droege, pers. comm.). A similar rapid rise in abundance of an introduced bee species with concomitant declines in related native species has been reported for native bumble bees in Japan, Argentina, and Chile following the introduction of non-native bumble bees for commercial pollination^12–14^. Our work adds to this understudied field and further highlights the need to monitor native populations when related exotic solitary bee species enter new ecosystems.

The reasons for the success of *Osmia taurus* relative to that of native *Osmia* are not known, but there are several plausible mechanisms: competition for resources, habitat changes favoring the introduced species, release from natural enemies in the introduced range, and concurrent introduction of novel diseases with the exotic species. If competition were the primary driver of declines between *O. taurus* and native species, then we would expect a gradient of change among native species based on their amount of niche overlap with *O. taurus*. In this case, *O. lignaria* would likely be the native species in greatest decline. It is the only native species in the same subgenus as both exotic species (subgenus *Osmia*), and it uses similarly-sized cavities as the exotic species^21,33^. *Osmia lignaria* is one of only two native species to emerge as early as *O. cornifrons* and O. *taurus* (Supplementary Methods, Supplementary Table S3), and it is the only native species in this study to also use mud as its primary brood cell partition material (the other native *Osmia* in this study use masticated leaves)^21,33,34^. In the United States, *O. lignaria* uses similar floral resources to the exotic *O. cornifrons*^19,35^, and although floral preferences of *O. taurus* have yet to be studied in North America, it is known to collect floral resources from the same groups of plants as *O. cornifrons* in Japan^33^. Thus, *O. lignaria* would seem to have the greatest potential among the native species to be impacted by the introduction of these exotic species to their native habitat. Although the mean annual decline of *O. lignaria* is among the greatest in magnitude of the native species analyzed, its confidence intervals overlap with all other native species (Fig. 2, Table 1), indicating that ecological factors beyond competition are likely playing a role in native species declines.

Another possible mechanism is that of disease spread from exotic mason bee species to native mason bees. Introduction of exotic species along with their diseases can dramatically reduce the population of native species that are closely related or functionally similar^3,36,37^. One of the main diseases affecting the bee genus *Osmia* worldwide is chalkbrood^38^, caused by a group of fungi in the genus *Ascosphaera*. Several species of *Ascosphaera* are native to North America, but a recent study in Ithaca, New York (U.S.A.) found that the exotic *O. cornifrons* was harboring a Japanese species of *Ascosphaera* (*A. naganensis*), which it had apparently brought from its native range to the introduced range^39^. The pathogenicity of this *Ascosphaera* species is still unclear, as some *Ascosphaera* are saprophytic rather than parasitic and many other *Ascosphaera* species have not been studied^40^. It is also unknown to what extent this fungal species has moved from its exotic host into native hosts, but it remains an important avenue of study. In addition to *Ascosphaera naganensis*, the parasitoid wasp *Monodontomerus osmiae* Kamijo, known to attack *O. taurus and O. cornifrons* in Japan^41,42^, was recorded in North America in 2002 in Silver Spring, Maryland^43^. Its abundance and impact on native *Osmia* in the region, and how it came to be introduced into North America, however, have yet to be discerned. Similarly, the palearctic spider beetle *Ptinus sexpunctatus* Panzer, was first detected in North America in 2003 in the nests of *Osmia lignaria*^44^. It is known to be very destructive to *Osmia* nests in Europe and may represent a new, broad threat to North American *Osmia*.

Reduced pressure from natural enemies may be another likely pathway of successful invasion by exotic species^2,45^ and has been documented for cavity nesting bees in North America^46^. No parasite studies have been carried out for *Osmia taurus* in North America, so it is currently unknown if this mechanism contributes to its successful proliferation. In its native range in Japan, *O. taurus* and *O. cornifrons* are associated with natural enemies across eight orders of arthropods^33,47^. In North America, five nest associates of *O. cornifrons* have been reported, four of which are congeners to typical nest associates of *O. cornifrons* in Japan: *Chaetodactylus* (parasitic mites), *Monodontomerus* (parasitoid wasps), *Ptinus* (spider beetles), and *Tribolium* (flour beetles). The fifth associate is in the genus *Tricrania* (Meloidae), which is endemic to North America, but a meloid species in the genus *Meloe* is reported to attack *O. cornifrons* in Japan^33^. Thus, similar kinds of natural enemies were already present when *Osmia taurus* arrived, although we don't know the extent to which the native enemies are attacking *O. taurus*. One reason *O. cornifrons* may have been selected for import into the United States instead of *Osmia taurus* was because *O. taurus* experienced high levels of natural enemy attack in Japan^33,48,49^. If *O. taurus* is able to escape the frequency of enemy attack it experiences in its native range, it may exhibit higher reproductive output in an introduced environment with fewer natural enemies. Future studies should examine whether *O. taurus* is suffering less from natural enemies in its introduced range compared with native *Osmia* and thereby gaining an advantage over potential competitors.

Abiotic factors such as climate change may favor the proliferation of some species over others, but in the current study there is no evidence for habitat changes which favor the introduced species while disfavoring native species. However, the faster spread of *Osmia taurus* than *Osmia cornifrons* may reflect broader climatic tolerance in the introduced range. In Japan, where both species are native, *O. cornifrons* is restricted to the central and northern parts of the country, whereas *O. taurus* occurs throughout. Professor Yasuo Maeta^33^ hypothesized that the restricted distribution of *O. cornifrons* in Japan may reflect developmental failure in the southern and coastal climates: either, the hot summer prevented successful prepupal development, or the warm fall failed to induce overwintering diapause in time. *Osmia cornifrons* was selected for introduction into the United States due in part to a climatic match between its native range and that of temperate North America^19^. If *O. taurus* is able to tolerate a wider variety of climate conditions than *O. cornifrons*, then it may be poised to spread much further in its exotic range, particularly in warmer areas, than *O. cornifrons*. Range expansions or shrinkages in native *Osmia* species due to a warming climate in the Mid-Atlantic United States have yet to be evaluated but remain crucial for fully understanding cause(s) of native *Osmia* declines.

Another finding of note in our study was that the exotic *O. cornifrons* did not exhibit any significant changes in raw abundance over the thirteen-year period, and it is unclear if the lack of a significant trend indicates stability over time or lack of precision in estimates due to spatial and temporal variability. One factor that may contribute to its variability is its continual management and release into the environment as an agricultural pollinator of fruit trees in the Mid-Atlantic United States^50,51^. Although we are not aware of the release of commercially-managed *O. cornifrons* directly at any sites in our dataset, the regional production, protection, and redistribution of *O. cornifrons* could result in locally supplemented populations, providing a competitive advantage against native species and a buffer against the fast-spreading *O. taurus*.

This study takes advantage of an existing bee monitoring program that provides substantial documentation of bee populations over a large geographic area with nearly two decades of sampling made possible by citizen science program paticipants. Although *Osmia* are captured at lower rates than many other groups of bees using pan-trapping^52^ and thus may produce estimates with less precision than for more abundantly captured groups, this combined dataset provides the most information about *Osmia* abundances at this scale in North America to date. An affordable, general collecting protocol replicated over time and space with the help of many individuals such as citizen science program participants can provide a wealth of data, including information on changes in species abundance over time and exotic species detection and spread. Such monitoring is needed in order to recognize species declines in time to study their causes as well as prevent their loss from the landscape.

## Supplementary information

Supplementary Information 1.

Supplementary Information 2.

## Data Availability

The code used to produce the results and figures in this paper is available from the corresponding author upon request. The mixed models in this study were run using Statistical Analytics Suite (SAS) 9.4, a commercial software product.

## Data availability

All datasets for the main and supplementary analyses are available as Supplementary Information.
