# Supplementary material for: Decline of six native mason bee species following the arrival of an exotic congener
Source: Sci Rep. 2020 Oct 30;10:18745. doi: 10.1038/s41598-020-75566-9 (PMC7599227; doi:10.1038/s41598-020-75566-9)
Supplement: Supplementary file 2 — Supplementary Information 2. [file 41598_2020_75566_MOESM2_ESM.pdf]

Supplementary Information for:

Decline of six native mason bee species following the arrival of an exotic congener

Kathryn A. LeCroy[1]*, Grace Savoy-Burke[2], David E. Carr[1], Deborah A. Delaney[2], and T'ai H. Roulston[1]

1. Department of Environmental Sciences, University of Virginia, Charlottesville, VA USA
2. Department of Entomology and Wildlife Biology, University of Delaware, Newark, DE USA

**Corresponding author email:* kalecroy@gmail.com

Supplementary Methods

Analysis of intra-season sampling effort variation across time

Variation in sampling effort across years was accounted for in the full mixed models by setting "bowl days" as an offset variable for every cluster-year. However, if variation in sampling effort changed over these years within March to July, it could bias our estimations of increase or decline of catch frequencies based on the different phenology of each species. To examine variation of sampling effort over this scale, sampling events were coded into four time bins covering March to July (the springtime and early summer period in which all *Osmia* specimens were recorded), with each time bin split as evenly into days as possible from March 1 to July 24, the latest day of any sampling event in the time series (all time bins contained 36 days with the exception of the fourth time bin that contained 38 days). The response variable was the log-transformed sum of all bowl days per year within each time bin. Year, time bin, and their interaction are fixed effects.

Emergence timing among species

We compared relative emergence pattern among the eight most common species in the dataset. Of the 298 spatial-clusters used for overall analyses, there were 147 instances in which a spatial cluster had been sampled at least twice within the same year. Although the haphazard sampling pattern within spatial clusters does not yield certainty regarding the emergence time of any individual species on a given year, when clusters were sampled at least twice in the same year they present an opportunity to see if two species caught in the same cluster were first caught at different time periods. We looked at all species pairs independently, limiting the dataset to all cases in which those two species had been caught in the same cluster and that cluster had been sampled multiple times. We used a Wilcoxon signed rank test to determine if there was a significant difference in first detection between the two species. We used a Bonferroni correction to account for pairwise testing among all species, and refer to significance only for species pairs with a p-value < 0.0017 (Supplementary Table S3).

Landcover types sampled over time

Landcover type of sampling sites was assessed by analyzing percent cover within 200m of sampling sites using the remotely sensed National Land Cover Database 2011[1] in ArcMap 10.6.1[2]. Landcover types were grouped into five categories: Forest (deciduous, conifer, mixed forest, and woody wetlands); Developed (developed open space, developed low intensity, developed medium intensity, developed high intensity); Open Habitat (shrub/scrub and grassland/herbaceous); Agriculture (pasture/hay and cultivated crops); and Other (all other landcover types). Five simple regressions, one for each landcover type, were performed with percent landcover type as the response variable and "year" as the independent variable.

References

1. Homer, C. G., Dewitz, J. A., Yang, L., Jin, S., Danielson, P., Xian, G., Coulston, J., Herold, N. D., Wickham, J. D., & Megown, K. (2015). Completion of the 2011 National Land Cover Database for the conterminous United States - Representing a decade of land cover change information. *Photogrammetric Engineering and Remote Sensing* 81(5), 345-354. https://cfpub.epa.gov/si/si_public_record_report.cfm?dirEntryId=309950

2. ESRI (Environmental Systems Resource Institute). (2018) ArcMap 10.6.1. ESRI, Redlands, CA

Table S1. Summary table of *Osmia taurus* results including outlier from 2010 with 548 Osmia specimens captured in a single day. The estimated rate of increase remains positive (increase by 0.0037 from main analysis of *O. taurus*) and remains significant.

**Effect**	**Estimate (reverse i-link)**	**Standard Error Mean**	**DF**	**t**	**Pr > |t|**
year	1.1736	0.0370	105	**5.08**	**<.0001**

Table S2. ANOVA output of sampling effort over time. There was a significant difference among years in log-transformed bowl days (as expected and therefore accounted for in the full mixed models as the offset variable), the sampling effort was not significantly different across time bins, and there was no significant interaction effect between year and time bin. The finding of no significant difference in sampling effort across time bins, paired with the lack of a significant interaction between time bin and year gives us reasonable confidence that our analyses are not significantly biased by researcher-based shifts in sampling periods within the months spanning flight seasons of Mid-Atlantic species of *Osmia*. The failure to find a significant interaction allayed our concern that there was any systematic shift in sampling effort from 2003 through 2017.

Source	DF	MS	F	P
**Year**	**1**	**10.19**	**17.56**	**0.0001**
Month bin	3	1.34	2.32	0.0875
Year*Month bin	3	1.35	2.33	0.0862
Error	48	0.58		

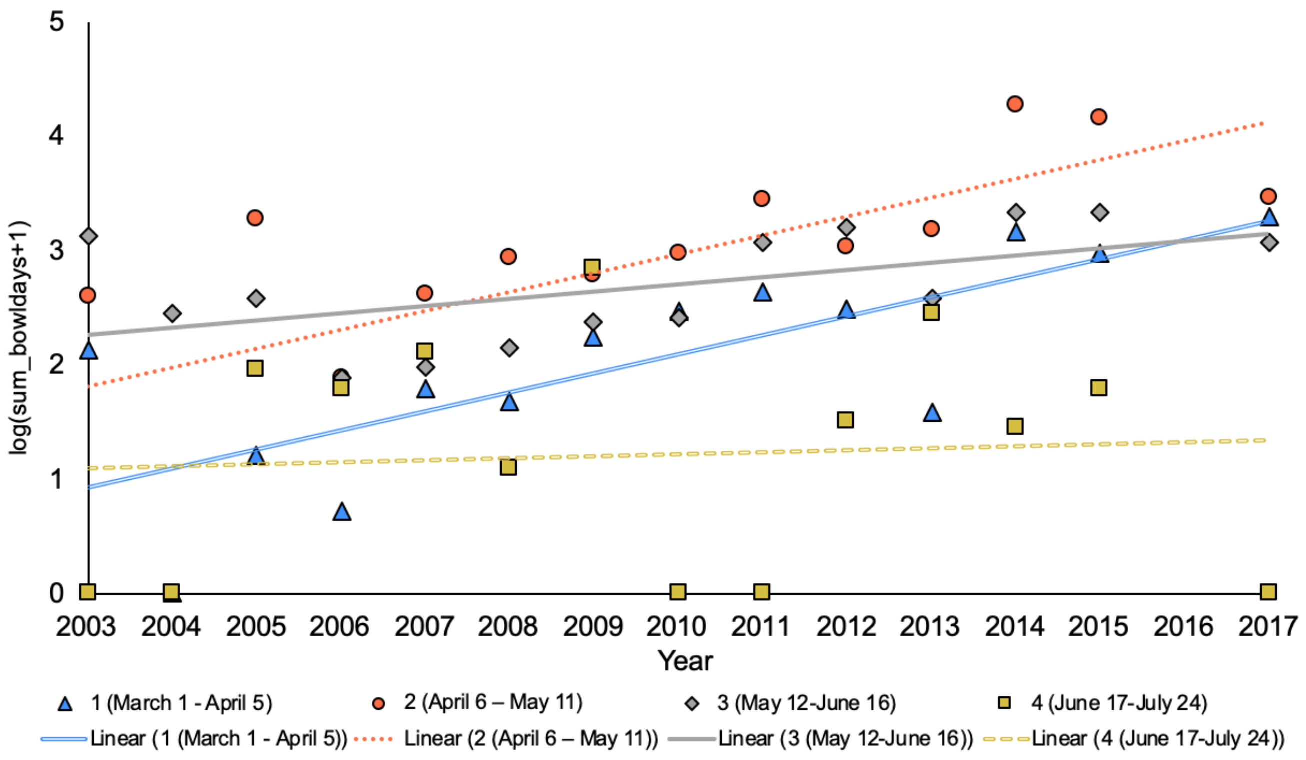

Figure S1. Log-transformed sums of bowl days over year, with lines of best fit for each time bin.

Table S3. P-values from pairwise comparison of *Osmia* species for the first capture date at shared site clusters on a given year. Statistical significance (*) determined using p=0.0017 following a Bonferroni correction of alpha = 0.05 for all site pairs. The exotic *Osmia taurus* was captured in pan-traps earlier in the spring than all native species except for *O. atriventris* and *O. lignaria*. The native *O. bucephala* and *O. georgica* were generally caught later than other species, but all species overlapped in collection times at some sites.

	O. bucephala	*O. georgica*	*O. collinsiae*	*O. lignaria*	*O. pumila*	*O. atriventris*	*O. cornifrons*	*O. taurus*
O. bucephala								
O. georgica	0.096							
O. collinsiae	1	0.792						
O. lignaria	0.371	0.281	0.09					
O. pumila	**< 0.001***	**<0.001***	0.005	0.55				
O. atriventris	0.02	0.008	0.15	0.306	0.41			
O. cornifrons	0.002	**<0.001***	0.17	0.484	0.127	0.07		
O. taurus	**<0.001***	**<0.001***	**<0.001***	0.023	**<0.001***	0.02	0.99	

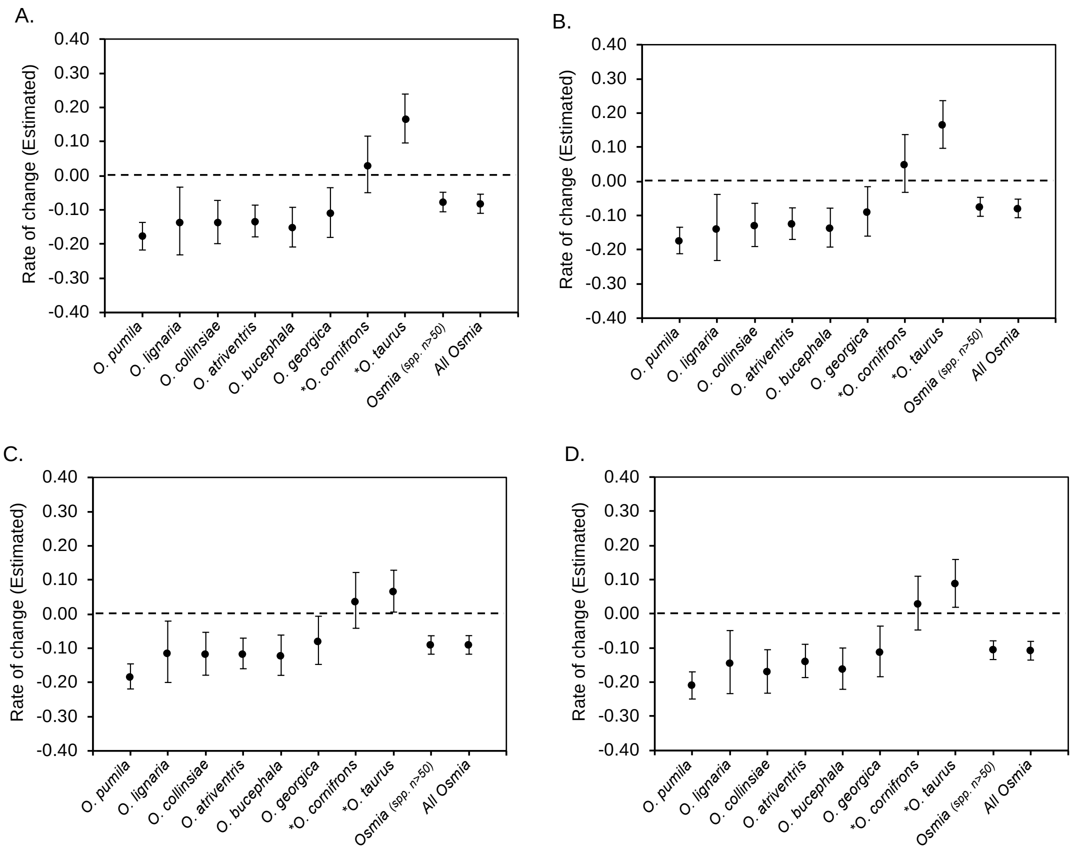

Figure S2. Estimated rates of yearly change for models of each species-specific and genus-wide analysis performed, with 95% confidence intervals. Estimates in original scale (reverse i-link) are displayed. Species labeled with an asterisk (*) are exotic to North America. A) results using 3 km clustering; B) results using 5 km clustering; C) results using 10 km clustering; D) results using 20 km clustering.

Table S4. Measure of Moran's I of the standardized residuals for each species-specific and genus-wide models used in main analysis. Significance is denoted as **** = < 0.0001; *** = < 0.001; ** = < 0.01; * = < 0.05.

Species	Moran's I
Osmia atriventris	0.0029
Osmia bucephala	-0.0267
Osmia collinsiae	0.0256
Osmia cornifrons	-0.0020
Osmia georgica	0.0291
Osmia lignaria	0.0201
Osmia pumila	0.0457*
Osmia taurus	0.0308
genus Osmia (n>50)	0.0880
genus Osmia (all)	0.0880

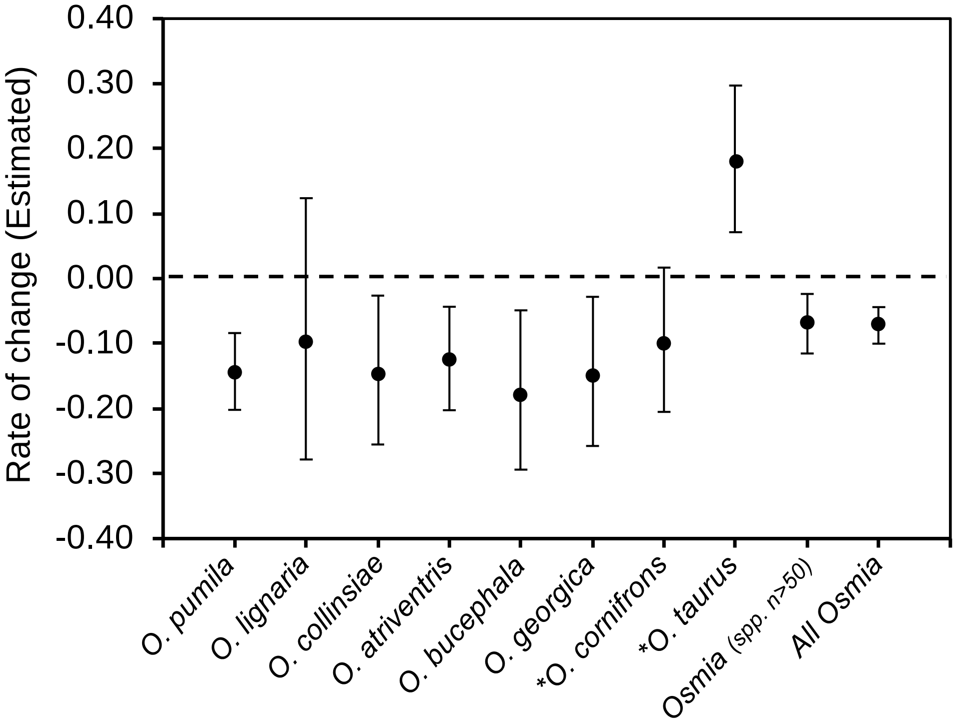

Figure S3. Only multi-sampled clusters used for analyses: xx number of locations. Estimated rates of yearly change for models of each species-specific and genus-wide analysis performed, with 95% confidence intervals. Estimates in original scale (reverse i-link) are displayed. Species labeled with an asterisk (*) are exotic to North America.

Table S5. Summary of analysis only including multi-sampled clusters. Estimates are given on original scale. NB = Negative Binomial distribution selected for analysis (as described in Methods section).

Species	Native/Exotic	*n*	Model	Estimated Mean Change Per Year	S.E.	P	Percent Mean Change Per Year
Osmia atriventris	Native	257	NB	**0.8735**	**0.0400**	**0.0040**	**-12.65**
Osmia bucephala	Native	114	NB	**0.8194**	**0.0615**	**0.0093**	**-18.06**
Osmia collinsiae	Native	63	NB	**0.8515**	**0.0576**	**0.0193**	**-14.85**
Osmia cornifrons	Exotic	384	NB	0.8990	0.0558	0.0894	-10.1
Osmia georgica	Native	155	NB	**0.8495**	**0.0576**	**0.0180**	**-15.05**
Osmia lignaria	Native	41	NB	0.9006	0.1005	0.3500	-9.94
Osmia pumila	Native	745	NB	**0.8550**	**0.0298**	**<.0001**	**-14.5**
Osmia taurus	Exotic	1320	NB	**1.1788**	**0.0568**	**0.0009**	**+17.88**
genus Osmia (n>50)	Both	3079	NB	**0.9295**	**0.0231**	**0.0040**	**-7.05**
genus Osmia (all)	Both	5901	NB	**0.9275**	**0.0142**	**<.0001**	**-7.25**

Table S6. Regression metrics for each of the five landcover types analyzed over time in data series. There were no statistically significant regression relationships found for any percent landcover type change over years in the study.

Source	*B*	*SE B*	*t*	*p*	β
year vs. percent forest	0.1759	0.3833	0.4600	0.6466	0.0229
year vs. percent agriculture	0.1423	0.3088	0.4600	0.6451	0.0230
year vs. percent developed	-0.3280	0.2860	-1.1500	0.2521	-0.0572
year vs. percent open habitat	-0.0692	0.0716	-0.9700	0.3345	-0.0482
year vs. percent other	0.0790	0.1899	0.4200	0.6776	0.0208
